# 

**Published:** 1846-09

**Authors:** 


### CONTENTS

OF NO. III.

### ORIGINAL COMMUNICATIONS.

### ESSAYS AND CASES.

ART. I.—The History, Value, Uses, and Effects of Quinine and its various salts. By SAMUEL HENRY DICKSON, M.D., Professor of the Institutes and Practice of Medicine in the Medical College of the State of South Carolina. - - - - - - - - 185

ART. II.—A Case of Double Consciousness, with Remarks by Professor Caldwell. By B. F. BERKLEY, M.D. of Moorfield, Ohio, in a letter to Prof. C. - - 204

ART. III.—Observations on the Blood in Asthma, Epilepsy, Hydro-Pericardium, and Bilious Fever. By J. R. BUCK, M.D., of Louisville, Ky. - - - - 211

ART. IV.—Notes on Medical Matters and Medical Men in London. By DAVID W. YANDELL, M.D. - 217

### REVIEWS.

ART. V.—Animal Chemistry with reference to the Physiology and Pathology of Man. By Dr. J. FRANZ SIMON, Fellow of the Society for the advancement of Physiological Chemistry, etc., etc. Translated and edited by GEORGE E. DAY, M.A.&L.M., Cantab., Licentiate of the Royal College of Physicians. - - 236

ART. VI.—Observations on Remittent Fever as it occurs in the Southern part of Alabama. By WILLIAM M. BOLING, M.D., of Montgomery, Ala. - - - 255

SELECTIONS FROM AMERICAN AND FOREIGN JOURNALS.

Alkalies a Remedy in Cutaneous Diseases. - - - 257
Case of Placenta Prævia. - - - - - - 258
On Chlorosis in Adults. - - - - - - - 259
Dr. Forbes and Homœopathy. - - - - - - 260
Employment as a Cause of Scrofula. - - - - 265
Drug Shop of Galen. - - - - - - - - 266
Mode of conducting a Pathological Analysis of the Blood. - 267
New Remedy for Strangury. - - - - - - 268
Fruitfulness of Foreigners in Boston. - - - - 268
Sketch of Dupuytren. - - - - - - - 269

ORIGINAL INTELLIGENCE.

Foreign Correspondence. - - - - - - - 273
Health of Louisville. - - - - - - - 274
Louisville Law School. - - - - - - - 274
Louisville Summer School of Medicine. - - - - 275
Errata. - - - - - - - - - - 275
Facts from Correspondents. - - - - - - 276

